# *N*-acyl homoserine lactones lactonase est816 suppresses biofilm formation and periodontitis in rats mediated by *Aggregatibacter actinomycetemcomitans*

**DOI:** 10.1080/20002297.2023.2301200

**Published:** 2024-01-07

**Authors:** Zelda Ziyi Zhao, Junmin Wang, Xinpai Liu, Zezhi Wang, Xianyu Zheng, Wuli Li, Tianfan Cheng, Jing Zhang

**Affiliations:** a Stomatological Hospital and College, Key Lab. of Oral Diseases Research of Anhui Province, Anhui Medical University, Hefei, Anhui, China; b Division of Periodontology & Implant Dentistry, Faculty of Dentistry, The University of Hong Kong, Hong Kong SAR, China

**Keywords:** Acyl-homoserine lactones, *Aggregatibacter actinomycetemcomitans*, biofilm, virulence factor, periodontitis

## Abstract

**Aims:**

The current study aimed to explore the adjuvant therapeutic effect of N-acyl homoserine lactones (AHLs)-lactonase est816 on *Aggregatibacter actinomycetemcomitans (A. actinomycetemcomitans)* biological behaviors and periodontitis progression.

**Methods:**

The inhibitory properties of est816 were detected by live/dead bacterial staining, scanning electron microscope (SEM), crystal-violet staining and reverse-transcription quantitative PCR (RT-qPCR). Biocompatibility of est816 on human gingival fibroblasts (HGFs) and human gingival epithelial cells (HGEs) was evaluated by CCK8 and ELISA. The ligature-induced periodontitis model was established in rats. Micro computed tomography and immunohistochemical and histological staining served to evaluate the effect of est816 on the prevention of periodontitis *in vivo*.

**Results:**

est816 significantly attenuated biofilm formation, reduced the mRNA expression of cytolethal distending toxin, leukotoxin and poly-N-acetyl glucosamine (PNAG) and downregulated expressions of interleukin-6 and tumor necrosis factor-α with low cell toxicity. In vivo investigations revealed est816 decreased alveolar bone resorption, suppressed matrix metalloproteinase-9 expression and increased osteoprotegerin expression.

**Conclusion:**

est816 inhibited *A. actinomycetemcomitans* biofilm formation and virulence release, resulting in anti-inflammation and soothing of periodontitis in rats, indicating that est816 could be investigated in further research on periodontal diseases.

## Introduction

Periodontitis is a chronic and progressive infection by a bidirectional imbalance between the inflammatory response and oral microbiota. The abnormal microenvironment also results in host response and the uncontrolled inflammation, further aggravating loss of the periodontal ligament and damage to the surrounding alveolar bone [1]. One of the causative agents in the pathogenesis of periodontitis is *A. actinomycetemcomitans*, a highly pathogenic bacterial species of the oral microbiome [2]. Complete removal of *A. actinomycetemcomitans* biofilm by mechanical debridement is difficult due to a small percentage of *A. actinomycetemcomitans* in periodontal pockets still survive and reattach rapidly to form new biofilms, thus leading to recurrent periodontitis [3]. Systemic antibiotics effectively kill planktonic *A. actinomycetemcomitans*, while antibiotic has limited effect on biofilm cells. Besides, long-term exposure to systemic antibiotics increases the risk of bacterial resistance [4]. Therefore, it is necessary to find an ideal antibiofilm agent for promoting antibacterial activity.

Biofilm formation and maturation are mainly regulated by quorum sensing (QS), which depends on the synthesis of small signal molecules that regulate virulence factor expression and biofilm development in a density-dependent manner [5]. Acyl-homoserine lactones (AHLs) are important QS-related signaling molecules that are mainly produced by Gram-negative bacteria [6]. AHLs have been isolated from several oral pathogens in dentine caries and dental plaque [7]. Besides, AHLs are critical for the attachment and colonization of bacterial to host surface which initiate and drive a pathophysiologic inflammatory response. Exogenous AHLs and their analogs were previously shown to affected biofilm formation and slowed *Porphyromonas gingivalis* growth, which highlighted the critical role of AHLs-mediated QS in oral microbiota and biofilm formation [8]. Furthermore, exogenous AHLs promoted biofilm formation and regulated the transcriptional network involved in virulence and stress tolerance in *Enterococcus faecalis* strains, which raised an insight into the Gram-positive bacteria among oral polymicrobial communities [9]. Therefore, suppression of AHLs as a treatment target may be a promising adjuvant treatment strategy for biofilm-associated oral infectious diseases.

AHLs are composed of homoserine lactone rings linked by an amide bond to a fatty acid (4 to 20 carbons) [10]. AHL-lactonases are a kind of quorum quenching enzymes that hydrolyze the lactone ring of AHLs and decrease the concentration of AHLs, disrupting AHLs-mediated QS during biofilm development [11]. Many successful *in vitro* and *in vivo* studies have indicated the role of AHL-lactonases in reducing the production of virulence factors and motility and in inhibiting biofilm formation of bacterial pathogens, such as *Pseudomonas aeruginosa* and *Acinetobacter baumannii* [12,13]. A series of studies focused on the AHL-lactonase Aii20j previously verified the inhibitory impacts on biomass production and taxonomical composition of cariogenic biofilms from clinical supragingival plaque samples as well as on the polymicrobial biofilm formation from patients with periodontal disease [14]. However, the application of AHL-lactonases in the oral field still remains limited for the technological deficiency of AHL detection in the clinical samples and the lack of bacterial virulence mechanism research among the periodontal pathogens [15,16].

In a previous study, we cloned est816, a novel AHL-degrading enzyme with high hydrolytic activity against all types of medium- and long-chain AHLs (C_4_-C_12_). We found that est816 has excellent stability at pH 5.0–9.0 and below 50°C, and it is suitable for oral application according to the pH and temperature of buccal cavity [17]. Owing to the evidence of AHL family signaling molecules produced by periodontal bacterial communities in laboratory samples and from clinical samples, it raised a question whether AHL-mediated QS mechanism played a role in the biological behavior of *A. actinomycetemcomitans* [15]. Therefore, the aim of the current study was to evaluate whether AHL-lactonase est816 could prevent *A. actinomycetemcomitans* biofilm formation and virulence factor release *in vitro* and to further investigate its inhibitory effect on the recurrent progression of periodontitis in rats. The study results offer new perspectives on AHL-lactonases in the prevention and adjuvant treatment of oral infectious diseases.

## Materials and methods

### Bacterial strains and reagents

*A. actinomycetemcomitans* ATCC43717 (BeNa Culture Collection, Beijing, China) was used to investigate bacterial biofilm formation. The lyophilized *A. actinomycetemcomitans* was mixed with sterile water, 200 μL of which was then coated evenly on Columbia Blood Agar (Landbrige Co., Jiangsu, China). Afterwards, it was cultured in an anaerobic environment at 37°C and 5% CO_2_ for 48 h. A single colony as the inoculum was sub-cultured in brain heart infusion (BHI) medium (3.6% brain-heart broth, 0.5% yeast extract, 0.5% hemin, 0.1% vitamin K1) (Landbrige Co., Jiangsu, China) for all experiments. Forty-eight hours later, the culture was diluted to the appropriate bacterial cell density (1.0 × 10^7^ CFU mL ^−1^) with BHI medium for subsequent biofilm development.

### Purification of est816

est816 was prepared according to previous instructions [18]. After sonication disruption, the supernatant of the *E. coli* BL21 (DE3) cells which expressed the putative AHL-lactonase gene was collected by centrifugation (13,000 × g, 10 min) at 4 ℃. The sample was then loaded onto a Ni-NTA His·Bind column and was washed with binding buffer and washing buffer (0.5 M NaCl, 60 mM imidazole, 20 mM Tris – HCl, pH 7.9). Finally, the bound protein was eluted with eluting buffer (1 M imidazole, 0.5 M NaCl, 20 mM Tris-HCl, pH 7.9). The fractions containing the recombinant protein est816 were collected. One-unit enzymatic activity of est816 was defined as the amount of enzyme that produced 1 µmol of ρ-nitrophenol per minute, according to the substrate specificity of esterases/lipases [19].

### Biofilm assessment

A total of 500 μL of *A. actinomycetemcomitans* suspension (1.0 × 10^7^ CFU mL^−1^) were seeded into 48-well plates containing a sterile glass coverslip co-cultured with 500 μL of est816 at different concentrations of 6, 12, or 24 U mL^−1^, and with the hyperthermia-inactivated est816 solution at the initial concentration of 24 U mL^−1^, anaerobically for 48 h at 37°C. A confocal laser scanning microscope was used to evaluate the effects of est816 on biofilm growth. The dead and live bacterial cells were stained according to the manufacturer’s instructions (LIVE & DEAD Baclight Bacterial Viability Kit, L7012 Molecular Probes, Invitrogen, USA). The mixture solution of SYTO 9 (3.34 mM) and PI (20 mM) in a 1:1 proportion was diluted 150-fold in sterile PBS (pH 7.4), to the final working concentrations of 11 μM for SYTO 9 and 66 μM for PI. 50 μL of the staining solution was added onto each biofilm, and samples were incubated for 15 min at room temperature protected from light prior to image acquisition. The fluorescent images were observed by confocal laser scanning microscope (LSM880, Zeiss, German) at × 20 magnification. For the scanning electron microscope (SEM) assay, the samples in each group were washed with PBS for 10 min and then dehydrated in serial ethanol (30%, 50%, 70%, 80% and 90%) for 15 min, respectively, followed by an adequate dehydration in three changes of 100% ethanol. Next, the samples were dried in vacuum, coated with gold, and then analyzed using an environmental SEM. Three biofilms and three fields in each group were observed. Crystal-violet staining assay was performed to quantitatively examine biofilm mass. The coverslips were gently washed thrice with phosphate-buffered saline (PBS, Solarbo, Jiangsu, China) to discard any unattached bacterial cells. Subsequently, the biofilms attached to coverslips were fixed by methanol for 15 min and air-dried for 10 min. Then, the samples were stained with 200 μL of 1% crystal violet for 20 min, rinsed with PBS thrice and dried for 20 min. Next, crystal violet-stained biofilms were dissolved by absolute ethanol to measure spectrophotometrically at OD_590_. The lethality of est816 in planktonic *A. actinomycetemcomitans* was measured. The *A. actinomycetemcomitans* overnight culture was diluted to OD_600_ value at 0.2, then inoculated with additional est816 at concentrations of 6, 12, or 24 U mL^−1^ and the inactivated est816 for treatment. The bacterial density at OD_600_ was measured to obtain the growth curve.

### Expression of virulence genes

RT-qPCR assay was performed to evaluate the effects of AHLs-lactonase est816 on the expression levels of leukotoxin (*lktA*), cytolethal distending toxin (*cdtB*) and PNAG synthesis gene (*pgaA*) by *A. actinomycetemcomitans* both in planktonic culture and in biofilm condition. RNA was extracted from *A. actinomycetemcomitans* pre-treated with or without different concentrations of est816 according to the manufacturer’s instructions (RNA protect Bacteria Reagent 76,506, Qiagen, German). Then, cDNA was synthesized according to the manufacturer’s instructions (PrimeScript RT reagent Kit, Takara, Japan). The sequences of PCR primers used in this study are shown in Supporting Information Table 1. The provided reference gene in quantitative analysis of *A. actinomycetemcomitans* mRNA expression was 16S rRNA. The PCR reaction parameters were as follows: initial denaturation step of 95°C for 30 s, and another 40 cycles of melting step at 95°C for 3 s, followed by 60°C for 30 s. All samples were tested in triplicate (technical replicates of RT-qPCR on the same RNA extract).

### Evaluation of cell toxicity of est816

To evaluate the cell toxicity of est816, HGFs and HGEs were seeded in 96-well plates at 5 × 10^3^ cells well^−1^ and cultured for 24 h to cell adherence. The medium was then removed, and the plates were supplemented with culture medium followed by the different concentrations of est816 at a ratio of 1:1, in which the final working concentrations of est816 reached to 6, 12 and 24 U mL ^−1^. For the control group, medium and PBS were added at the same proportions. After 1, 3, 5, and 7 days of incubation, 100 mL of diluted CCK-8 reagent was added, and the plates were cultured for 2 h prior to analyzing optical density at 450 nm.

### Effects of biofilm supernatants on the production of IL-6 and TNF-α

ELISA assay was utilized to determine the protein levels of interleukin-6 (IL-6) and tumor necrosis factor-α (TNF-α) in HGFs and HGEs supernatants, respectively (ELISA Kits, Solarbio, Beijing, China). The groups were divided into the *A. actinomycetemcomitans* group, est816+*A. actinomycetemcomitans*, and control groups. Two types of cells were stimulated by supernatants of *A. actinomycetemcomitans* biofilm (*A. actinomycetemcomitans* group), of biofilm pre-treated with 12 U mL^−1^ of est816 (est816+*A. actinomycetemcomitans* group), and of BHI medium mixed with the hyperthermia-inactivated est816 solution (initial concentration of 12 U mL^−1^) (control group). The supernatants of cell culture were collected at 3 h and 12 h, respectively. Unstimulated cells were served as control which received the equivalent amounts of sterile BHI medium as the biofilm supernatants. IL-6 and TNF-α in the supernatants were measured.

### Effects on periodontitis progression in rats

#### Animals

Thirty male Sprague-Dawley (SD) rats aged 6 to 8 weeks (160–200 g) were purchased (Animal Experimental Center of Anhui Medical University, Anhui, China). The protocol for all experimental procedures was conformed to the ARRIVE guidelines, the Use of Laboratory Animals of the National Institutes of Health. It was approved by the animal ethics committee of Anhui Medical University, China (Protocol No. LLSC20230821). All SD rats previously received azithromycin (10 mg/500 mL) for 4 days to reduce the original oral flora before the periodontitis model commenced in a controlled-temperature environment (22 ± 2°C) [20]. This treatment was followed by 7-day antibiotics-free period. The mice were randomly assigned to one of the following six experimental groups (*n* = 5):

Group 1: No ligation + normal saline, 1-month treatment;

Group 2: Ligation + *A. actinomycetemcomitans* suspension, 1-month treatment;

Group 3: Ligation + *A. actinomycetemcomitans* suspension + est816 solution, 1-month treatment;

Group 4: No ligation + normal saline, 2-month treatment;

Group 5: Ligation + *A. actinomycetemcomitans* suspension, 2-month treatment;

Group 6: Ligation + *A. actinomycetemcomitans* suspension + est816 solution, 2-month treatment.

A sterile nylon thread ligature was tied to the cervical area of the bilateral maxillary first molars for 2 weeks to establish a periodontitis model. The gingival pouch of the ligation area in the *A. actinomycetemcomitans* and est816 + *A. actinomycetemcomitans* groups were treated with 0.15 mL of 1.0 × 107 CFU mL^−1^ *A. actinomycetemcomitans* suspension and a mixture of *A. actinomycetemcomitans* and 12 U mL^−1^ est816 every 4 days, respectively. Rats treated with physiological saline without ligature served as the control group. Samples were collected at 1 and 2 months.

#### Microcomputed tomography (micro-CT) measurement

At 1- and 2-month treatment, rats were euthanized. Maxillae were dissected, fixed with 10% buffered formalin for 24 h and stored in 70% ethanol until they were scanned by micro-CT (Model 1172; SkyScan, Kontich, Belgium). Images were analyzed by CT Analyzer software (Version 1.15.4.0+; SkyScan, Kontich, Belgium). To measure alveolar crest resorption, three sagittal points were chosen for each interproximal area and recorded as the shortest distance from the line connecting the cementoenamel junctions (CEJ) between the maxillary first and second molars to the alveolar bone crest (ABC), referred to CEJ-ABC (Supporting Information Figure 1a). Five furcated sites were recorded to measure and calculate the average values representing the resorption of alveolar bone at the bifurcation of the root (Supporting Information Figure 1b). For volumetric measurements, three-dimensional regions of interest (ROIs) included the alveolar bone between the four distal roots of the first molar and the mesiobuccal and mesiopalatal regions of the second molar, which was the main part of bone resorption after periodontal ligature (Supporting Information Figure 1c). The percentage of residual bone volume/total bone volume (BV/TV) within the ROI was regarded as BV/TV (*n* ≥ 3/group for all micro-CT analyses). Two technicians previously trained and calibrated, independently measured and averaged the results. Repeated measurement and personnel comparison of the measured samples showed no significant difference.

**Figure 1.**
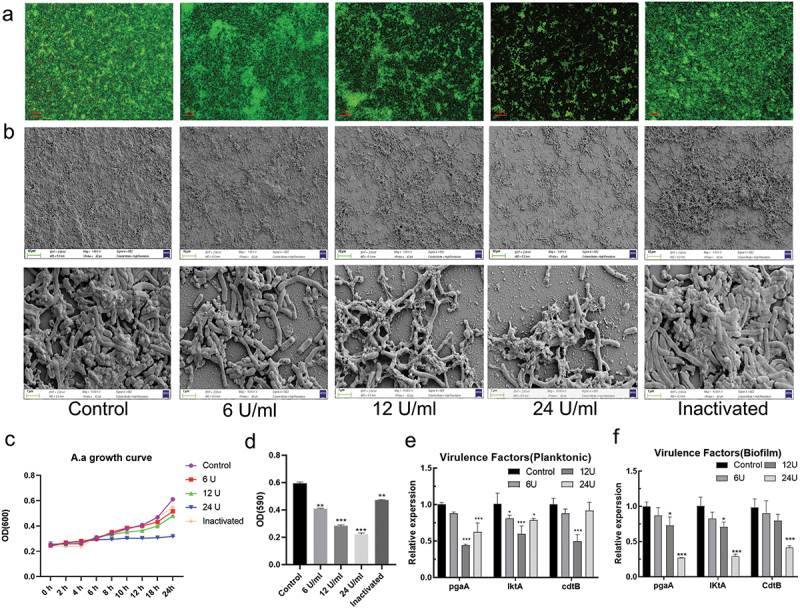
The effect of est816 on *A. actinomycetemcomitans* biofilm formation and virulence factor release. (a) confocal microscopy visualization of the effect of 6, 12, 24 U ml^−1^ and inactivated lactonase est816 on *A. actinomycetemcomitans* biofilm growth (original magnification × 20). (b) SEM images of *A. actinomycetemcomitans* biofilm pretreated with 6, 12, 24 U ml^−1^ and inactivated est816 (original magnification × 1000 and × 10,000). (c) The growth curve of planktonic *A. actinomycetemcomitans* cultured with 6, 12, 24 U ml^−1^ and inactivated lactonase est816. (d) The quantitative analysis of crystal violet staining measurement. (e-f) real-time PCR analysis of the effect of 6, 12 or 24 U ml^−1^ est816 on the expression of *pgaA*, *lktA* and *cdtB* of *A. actinomycetemcomitans* in planktonic form and biofilm condition (est816 groups were compared with the control group, *p < 0.05, **p < 0.01, ***p < 0.001). Data are the average of triplicate measurements, and error bars represent the standard deviation.

#### Histopathological and immunohistochemical analysis

After micro-CT scanning, all the maxillary tissues were decalcified in 10% ethylenediaminetetraacetic acid (EDTA) for 2 months, during which the decalcifying solution was replaced every 3 days. After decalcification, they were washed under running water for 15 min. The excess tissue was removed and cut into blocks of an appropriate size; only the first and second molars and the periodontal tissue around them were stored. After 2-month decalcification, the maxillae samples were prepared for the staining of hematoxylin-eosin, immunohistochemistry of matrix metalloproteinase-9 (MMP-9), receptor activator of the NF-κB ligand (RANKL) and osteoprotegerin. Results were analyzed by ImageJ software (Motic Medical 6.0; Motic, Xiamen, China). The details were noted in Supporting Information.

#### Statistics

The data were presented as the mean ± standard error of the mean. One-way analysis of variance (ANOVA) followed by Bonferroni’s test was applied to test significance between groups. *p* value < 0.05 indicated statistical significance.

## Results

### est816 inhibited A. actinomycetemcomitans biofilm formation

Confocal microscopy (Figure 1a) revealed that est816 inhibited *A. actinomycetemcomitans* biofilm formation after 48 h of treatment in a dose-dependent manner. The biofilm in both the control and the hyperthermia-inactivated est816 groups was notably dense and uniform. After treatment with est816, an inverse association was observed between the concentration of est816, and the biofilm attached to the surface of the glass slide. SEM yielded similar findings for the architecture of biofilms (Figure 1b), confirming that the biofilms of the control group and the hyperthermia-inactivated est816 group were also complete and dense and revealing that they had a certain thickness. After treatment with est816 at 6, 12, or 24 U ml^−1^, cultured biofilms gradually decomposed and were scattered in piled, networked, granular, and multilayered forms. It was also worth noting that the treatment of est816 at 24 U mL^−1^ marked a more significant difference (*p* < 0.05) on the growth curve in planktonic status of *A. actinomycetemcomitans* (Figure 1c). Crystal-violet staining (Figure 1d) showed that 6, 12, and 24 U ml^−1^ est816 exerted significant activity against *A. actinomycetemcomitans* biofilm mass in a concentration-dependent manner (*p* < 0.001).

### est816 decreased virulence genes of A. actinomycetemcomitans

The expression of *A. actinomycetemcomitans*-related virulence factors was analyzed to evaluate the effects of est816 on *A. actinomycetemcomitans* both in planktonic culture and in biofilm condition. We found that est816 significantly decreased the expression of *pgaA*, *lktA*, and *cdtB* compared with the control group. Figure 1e shows an overall trend of inhibition of the three virulence factors in planktonic bacteria culture affected by est816 of 6,12 and 24 U ml^−1^. The inhibitory effect of est816 on *pgaA*, *lktA* and *cdtB* of *A. actinomycetemcomitans* in the biofilm form was dose dependent (Figure 1f). At 12 U ml^−1^, est816 produced the greatest reduction on all the three factors. Therefore, the concentration of 12 U ml^−1^ was selected in further study.

### Cell toxicity of est816

**Figure 2.**
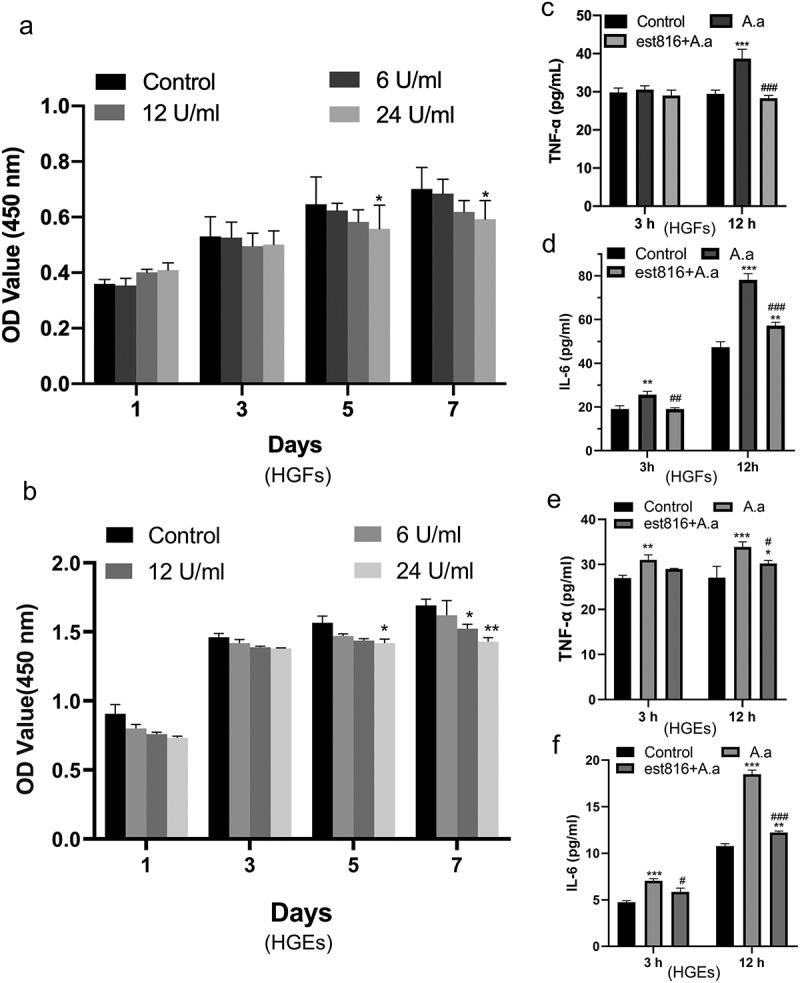
The cell toxicity and anti-inflammatory effects of est816. (a-b) CCK-8 results showing the proliferation of HGFs and HGEs treated with 6, 12, or 24 U ml^−1^ est816 for 1, 3, 5, and 7 days. (c-d) ELISA showing the protein expression of TNF-α (c) and IL-6 (d) in the supernatant of HGFs for 3 and 12 h. (e-f) ELISA showing the protein expression of TNF-α (e) and IL-6 (f) in the supernatant of HGEs for 3 and 12 h (*A. actinomycetemcomitans* group and est816 groups were compared with the control group, *p < 0.05, ***p < 0.001; est816 groups were compared with the *A. actinomycetemcomitans* group, ^#^*p* < 0.05, ^###^*p* < 0.001). Error bars of panels represent the standard deviation.

The CCK-8 assay was conducted to assess the effect of est816 on HGFs and HGEs proliferation. As shown in Figure 2(a,b) after 1, 3, 5, and 7 days of incubation, there was no significant difference in cell proliferation was present between the control group and the 6 U ml^−1^ est816 groups among the whole period. However, the proliferation of the two types of cells was inhibited by 24 U ml^−1^ of est816 on day 5 and day 7 and was statistically different from the control group. 12 U ml^−1^ of est816 had little effect on the viability of both HGFs and HGEs compared with the control group after 1, 3 and 5 days, except on day 7 which was statistically different (*p* < 0.05) from the control group.

### Effects of est816 on biofilm-induced production of IL-6 and TNF-α

ELISA showed that the expression of TNF-α (Figure 2c) and IL-6 (Figure 2d) in HGFs in both the *A. actinomycetemcomitans* and est816 + *A. actinomycetemcomitans* groups was not increased compared to that in the control group after 3 h of stimulation with biofilm supernatants. After 12 h of treatment, the production of TNF-α (Figure 2c) decreased significantly in the est816 + *A. actinomycetemcomitans* group compared to the *A. actinomycetemcomitans* group (*p* < 0.001), which showed no statistical difference to the control. Analysis of IL-6 (Figure 2d) showed that compared with the control group, the expression of IL-6 in the est816 + *A. actinomycetemcomitans* group was significantly lower than that in the *A. actinomycetemcomitans* group. The HGEs’ immune factor secretion levels were in accordance with the above results of the HGFs’. The secretion of TNF-α (Figure 2e) and IL-6 (Figure 2f) decreased significantly after 12 h of treatment in the est816 + *A. actinomycetemcomitans* group compared to the *A. actinomycetemcomitans* group (*p* < 0.05). Three-hour treatment of est816 alleviated the expression of IL-6 in the est816 + *A. actinomycetemcomitans* group which was significantly lower than that in the *A. actinomycetemcomitans* group (*p* < 0.05). These results revealed that est816 inhibited the biofilm toxicity to both HGFs and HGEs.

### est816 suppressed progression of periodontitis in rats

#### Micro-CT evaluation

**Figure 3.**
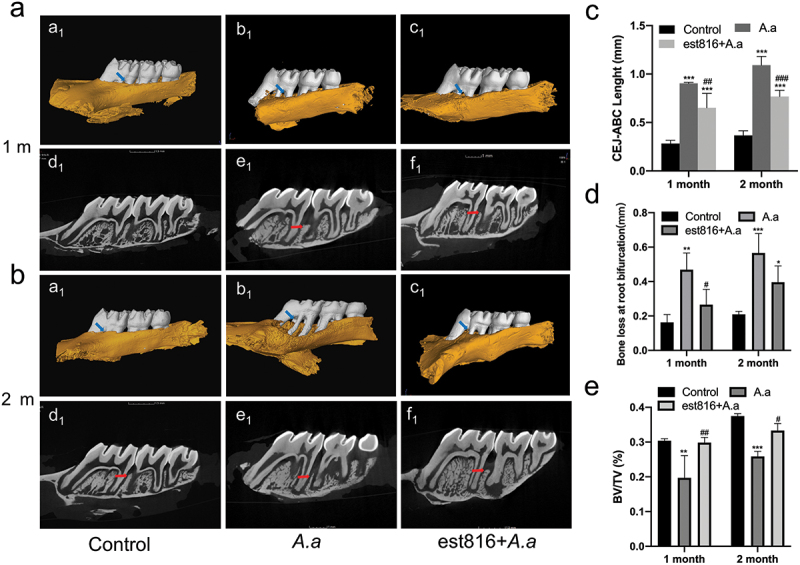
The results of micro-CT showing the effect of est816 on periodontitis in rats. (a-b) micro-CT exhibiting the bone loss at root bifurcation (a_1_, b_1_ and c_1_) and at alveolar bone crest (d_1_, e_1_ and f_1_) in the control, *A. actinomycetemcomitans*, and est816+*A. actinomycetemcomitans* groups after 1 and 2 months of treatment; (blue arrows point the region of root bifurcation; red arrows indicate the alveolar crest); (c-e) light and volume measurements showing alveolar crest resorption, bone loss of root bifurcation, and BV/TV of the DOI region in the control, *A. actinomycetemcomitans*, and est816+*A. actinomycetemcomitans* groups during 1 and 2 months, respectively (*A. actinomycetemcomitans* group and est816 + *A. actinomycetemcomitans* groups were compared with the control group, *p < 0.05, **p < 0.01, ***p < 0.001; est816 +*A. actinomycetemcomitans* group was compared with the *A. actinomycetemcomitans* group, ^*#*^*p* < 0.05, ^*##*^*p* < 0.01, ^*###*^*p* < 0.001).

Figure 3 (a,b) showed that compared with the control group, *A. actinomycetemcomitans* and est816 + *A. actinomycetemcomitans* groups presented bone loss at the root bifurcation and alveolar bone crest at 1 (Figure 3a) and 2 months (Figure 3b) of treatment. Notably, the *A. actinomycetemcomitans* group showed severer bone loss at the root bifurcation at 1 month (*p* < 0.05) (Figure 3a, a_1_–c_1_; Figure 3b, a_1_–c_1_, and Figure 3d) and more serious alveolar bone crest resorption at 2 months (*p* < 0.001) (Figure 3a, d_1_–f_1_; Figure 3b, d_1_–f_1_ and Figure 3c) than the est816+*A. actinomycetemcomitans* group. For the measurement of BV/TV in the fraction of ROI (Figure 3e), *A. actinomycetemcomitans* infection caused an evident decrease in bone tissue compared with the est816 + *A. actinomycetemcomitans* group (*p* < 0.01 at 1 month, *p* < 0.05 at 2 months). Favorably, there was no significant difference in BV/TV in ROI areas between the est816+*A. actinomycetemcomitans* and control groups. Additionally, the degree of bone resorption in the *A. actinomycetemcomitans* and est816+*A. actinomycetemcomitans* groups were time dependent.

#### Histopathology observation

**Figure 4.**
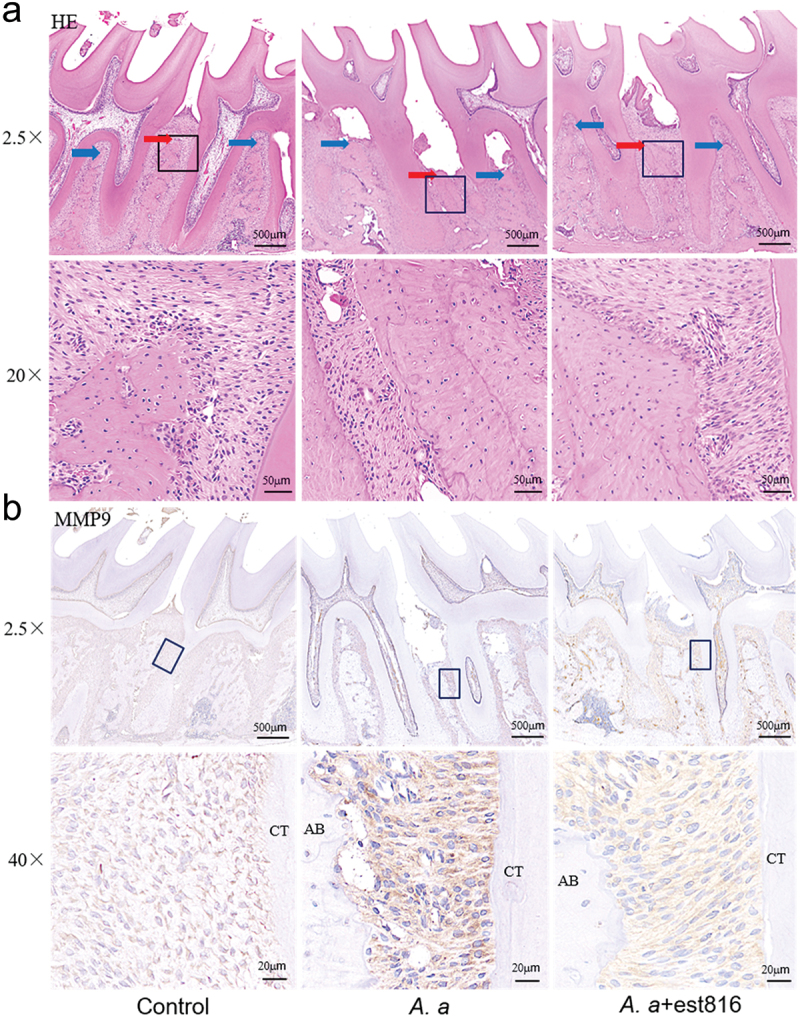
H&E staining and immunohistochemical analysis of MMP-9 in periodontal tissues. (a) H&E staining evaluating the inflammatory response of periodontal tissues in the region of the first and second molars of rats for 2 months (original magnification × 2.5 and × 20; blue and red arrows pointing root bifurcation and alveolar crest, respectively; the black boxes represent the magnification area). (b) Immunohistochemical analysis exhibiting MMP-9 expression in periodontal tissues in the region of the first and second molars of rats for 2 months; (original magnification × 2.5 and × 40; AB points to alveolar bone; CT points to cementum).

As shown in Figure 4a compared with the inflammatory status (state of the gingiva) and the periodontium structure (shape of the alveolar crest) of periodontal tissues between the first and second molars of the control group, in the same region of the est816+*A. actinomycetemcomitans* group, discrete cellular infiltration, preservation of alveolar bone, and intact cementum could be observed at 2 months. Conversely, rats subjected to *A. actinomycetemcomitans* presented more severe inflammatory cell infiltration and greater attachment loss and alveolar bone resorption coupled with destruction of the cementum at 2 months.

#### Immunohistochemistry assessment

**Figure 5.**
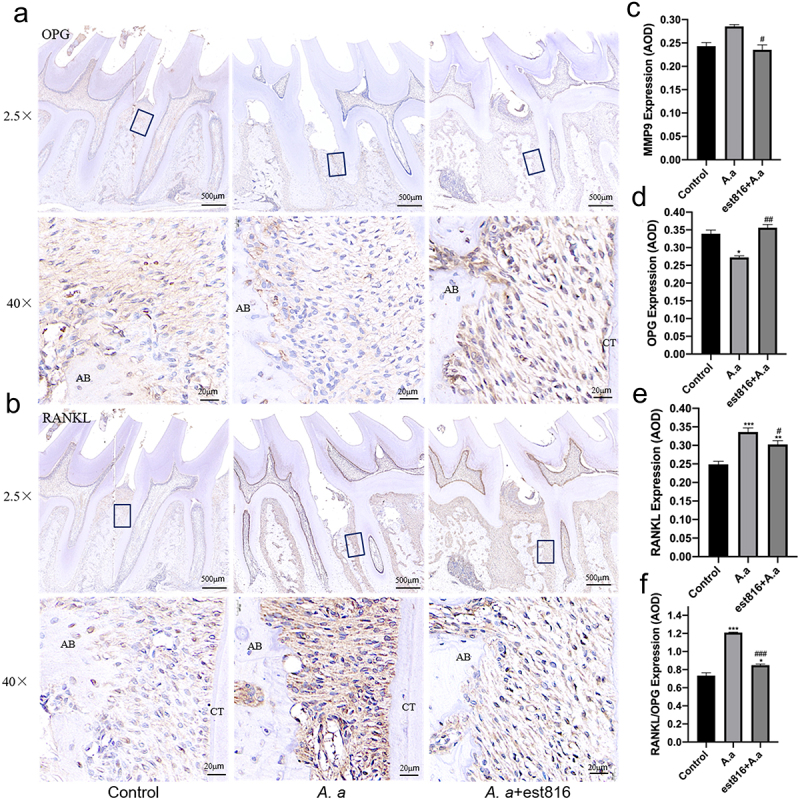
Immunohistochemical staining of OPG and RANKL and the averaged optical density (AOD) of bone loss-related markers. (a-b) immunohistochemical analysis exhibiting the OPG and RANKL expression of periodontal tissues of rats for 2 months; (original magnification × 2.5 and × 40; the black boxes represent the magnification area; AB, alveolar bone; CT, cementum); (c – f) the averaged optical density analysis showing the expression intensity of MMP-9, OPG, RANKL, and RANKL/OPG in periodontal tissues for 2 months in each group; (*A. actinomycetemcomitans* group and est816 + *A. actinomycetemcomitans* groups were compared with the control group, *p < 0.05, **p < 0.01, ***p < 0.001; est816 +*A. actinomycetemcomitans* group was compared with the *A. actinomycetemcomitans* group, ^*#*^*p* < 0.05, ^*##*^*p* < 0.01, ^*###*^*p* < 0.001). Error bars of panels (C, D, E and F) represent the standard deviation.

At 2 months, immunohistochemical analysis indicated that there was slight positive expression of MMP9 (Figure 4b) and RANKL (Figure 5b) in the control group. In the *A. actinomycetemcomitans* group, RANKL and MMP-9 were stronger and more positively stained than in the est816+*A. actinomycetemcomitans* group, while OPG (Figure 5a) was expressed intensely in est816+*A. actinomycetemcomitans*. The analysis of averaged optical density (AOD) showed that OPG expression was lowest, but the expression of MMP-9 and RANKL was highest in the *A. actinomycetemcomitans* group (Figure 5d). In contrast, the est816+*A. actinomycetemcomitans* group inhibited matrix degradation and bone loss-related marker MMP-9 (Figure 5c) and RANKL expression (Figure 5e). There was a significant increase in the RANKL/OPG expression ratio in the *A. actinomycetemcomitans* group (Figure 5f), while the est816 group dramatically reduced it compared with the *A. actinomycetemcomitans* group.

## Discussion

The production of AHLs by oral Gram-negative bacteria has been verified and shown to play an important role in regulating oral biofilm taxonomic composition and biofilm formation from cariogenic and subgingival samples [14,15]. AHL-lactonase can inhibit the development stage of biofilms and virulence factors by hydrolyzing the lactone rings of AHLs [21–25]. Compared with antibiotics, AHL-lactonase does not directly interfere with the growth of bacteria and has no toxicity to microbes [19]. However, few studies have explored the role of AHL-lactonase in the oral field, and its therapeutic effect on oral infectious diseases is scarce. The overall objective of this study was to explore, preclinically, the potential of AHL-lactonase on anti-biofilm and anti-inflammatory impact in preventing the progression of periodontitis. For this purpose, an *in vitro* biofilm model of *A. actinomycetemcomitans* was cultivated. In a mouse model of periodontitis induced by *A. actinomycetemcomitans* infection, the attenuation of alveolar bone loss by est816 was hypothesized, as well as the modulation of bacterial infection of periodontal tissues.

*A. actinomycetemcomitans* biofilm structure was less well formed after est816 treatment, which was revealed by live/dead bacterial staining, SEM and crystal staining, respectively. 6 and 12 U ml^−1^ est816 exerted a slight effect on *A. actinomycetemcomitans* growth, while est816 at 24 U ml^−1^ exerted an impact on the planktonic *A. actinomycetemcomitans*. Y. Asahi et al. reported *N*-acyl homoserine lactone analogues significantly exert their anti-biofilm activity by inhibiting the growth of *Porphyromonas gingivalis* cells and promoting the detachment of part of the biofilm [26]. Therefore, we speculated est816 indirectly inhibited *A. actinomycetemcomitans* growth by inhibiting tightly linked signaling molecules AHLs and impeding the bacterial communication, aggregation and biological characteristics. *A. actinomycetemcomitans* employs cytolethal distending toxin and leukotoxin to attack innate defense mechanisms and induce a pathophysiologic inflammatory response that would ultimately aggravate periodontal disease progression and accelerate bone loss [27–31]. Additionally, as an essential component in biofilms, PNAG promotes the adherence of bacteria to a surface and protects them from physical stresses and immune effectors [32]. The current study showed that est816 significantly suppressed the expression of the virulence genes and polysaccharide matrix in *A. actinomycetemcomitans* biofilm state and planktonic status as well. It partly due to est816 decreased *A. actinomycetemcomitans* self-aggregating, following less virulence factor release, ultimately inhibiting *A. actinomycetemcomitans* biofilm formation.

*A. actinomycetemcomitans* participates in the osteo-immunomodulatory effects of periodontitis pathogenesis. It effectively bypasses the gingival epithelium, including initial barriers and deeper subgingival tissues, and its virulence factors interact with host cells, thereby initiating an inflammatory response [33–35]. Proinflammatory cytokines with potent pro-resorptive functions, including IL-6 and TNF-α, are highly upregulated and thus induce osteoclast formation and bone resorption [36,37]. In this study, 12 U ml^−1^ of est816 effectively inhibited the inflammatory factors IL-6 and TNF-α produced by HGFs and HGEs with low cell toxicity. Briefly, the results revealed that 12 U ml^−1^ AHL-lactonase est816 had a potential anti-inflammatory effect with low cell toxicity.

Animal experimental periodontal disease was used to evaluate the inhibition effect of est816 on *A. actinomycetemcomitans* attachment and biofilm formation. The monobacterial infection of *A. actinomycetemcomitans* was introduced in this study. Owing to the *in vivo* condition that the polymicrobial consortium was consistent with the oral cavity environment, further exploration on the multi-species cultivation was needed to role of lactonase on animal models. The micro-CT results showed that the est816 + *A. actinomycetemcomitans* group retained much more alveolar bone than the *A. actinomycetemcomitans* group due to treatment of periodontitis with est816. The results suggested that est816 played an important role in preventing periodontitis recurrence in rats by resisting destructive pathologic impact on bone resorption.

Furthermore, bone loss-related markers were investigated, including RANKL, OPG and MMP-9. The RANKL/RANK signaling pathway modulates osteoclast formation, survival, and activation in normal bone modeling and in various pathologic conditions. The antagonistic mechanism of OPG against RANK protects bone from excessive resorption by combining to RANKL. Therefore, the ratio of RANKL/OPG plays a critical role in bone mass and strength. The upregulated RANKL/OPG ratio serves as a biomarker that denotes the occurrence of osteoclastogenesis followed by periodontitis [38,39]. MMP-9 modulates the extracellular matrix in periodontal tissue, and its selective expression can contribute to the acceleration of matrix degradation in periodontitis and other pathological conditions [40]. Analysis of the three markers was performed as a supplement to the proinflammatory cytokines in the anti-inflammation assay *in vitro* to improve the modulation mechanism of est816 on cellular and internal level. In the present study, the expression of RANKL/OPG was decreased and MMP-9 was weaker in the est816 + *A. actinomycetemcomitans* group, implying that it was not conducive to osteoclastogenesis and instead favored the inhibition of connective tissue destruction. These results were consistent with the micro-CT analysis, which showed that the est816 + *A. actinomycetemcomitans* group retained much more alveolar bone than the *A. actinomycetemcomitans* group due to periodontitis treatment with est816.

## Conclusion

est816 exhibited an obvious antibiofilm effect and exerted a positive effect against periodontitis progression in rats. These results indicated that AHL-lactonase could be investigated in further research on periodontal diseases as a prospective alternative for controlling oral infection.

## Supplementary Material

Supporting_Information_with_author_details.docx

## Data Availability

The data that support the findings of this study are openly available.
