# Supplementary material for: N-acyl homoserine lactones lactonase est816 suppresses biofilm formation and periodontitis in rats mediated by Aggregatibacter actinomycetemcomitans
Source: J Oral Microbiol. 2024 Jan 7;16(1):2301200. doi: 10.1080/20002297.2023.2301200 (PMC10773656; doi:10.1080/20002297.2023.2301200)
Supplement: Supporting_Information_with_author_details.docx [file ZJOM_A_2301200_SM6318.docx]

**Supporting information**

***N*-Acyl Homoserine Lactones Lactonase est816 Suppresses Biofilm Formation and Periodontitis in Rats Mediated by *Aggregatibacter actinomycetemcomitans***

Zelda Ziyi Zhao^a^, Junmin Wang^a^, Xinpai Liu^a^, Zezhi Wang^a^, Xianyu Zheng^a^, Wuli Li^a^, Tianfan Cheng^b*^, Jing Zhang^a*^

^a^ Stomatological Hospital and College, Key Lab. of Oral Diseases Research of Anhui Province, Anhui Medical University, Hefei, Anhui 230032, China

^b^ Division of Periodontology & Implant Dentistry, Faculty of Dentistry, The University of Hong Kong, Hong Kong SAR, China

***Corresponding author:**

Jing Zhang, PhD, Stomatological Hospital and College, Anhui Medical University, Meishan Road 69, Hefei, Anhui, China, Email: zhangjinglh817@126.com

 (e-mail can be published)

Tianfan Cheng, PhD, Division of Periodontology & Implant Dentistry, Faculty of Dentistry, The University of Hong Kong, Prince Philip Dental Hospital, 34 Hospital Road, Sai Ying Pun, Hong Kong SAR, China, Email: chengtfc@hku.hk

**Materials and methods**

**Evaluation of the degradation of AHLs by** **High-performance Liquid Chromatography (HPLC) Analysis**

HPLC was used to measure the degradation efficacy of est816 to AHLs commonly found in the oral cavity, which included *N*-octanoyl-DL-homoserine lactone (C_8_-HSL), *N*-dodecanoyl-L-homoserine lactone (C_12_-HSL), and *N*-(3-oxooctanoyl)-DL-homoserine lactone (OC_8_-HSL). It is processed as described previously. In brief, final concentrations of 1 mM C_8_-HSL, C_12_-HSL and OC_8_-HSL were prepared in pH 6.8 potassium phosphate buffer (50 mM) containing 5% methanol. Next, 10 mg of enzyme samples were added to 1 mL substrates to start the hydrolysis reaction at 30 ^°^C for 20 min. The control group was added elution buffer to substrates as a reference to consider the effect of nonenzymatic hydrolysis of C_8_-HSL, C_12_-HSL, and OC_8_-HSL.

**Determination of molecular mass**

The molecular mass of the denatured protein of est816 was determined by sodium dodecyl sulfate-polyacrylamide gel electrophoresis (SDS-PAGE). Proteins were stained with Coomassie brilliant blue G-250. The molecular mass of the enzyme subunit was estimated using protein marker as standards, rabbit muscle phosphorylase B (97,200 Da), bovine serum albumin (66,409 Da), ovalbumin (44,287 Da), carbonic anhydrase (29,000 Da), Soybean Trypsin Inhibitor (20,100 Da) and Hen egg white Lysozyme (14,300 Da) according to the previous detection methods [1].

**Evaluation of** **Biocompatibility of est816**

**Cell Cultures**

Human gingival fibroblasts (HGFs) were purchased (BeNa Culture Collection, Beijing, China). Human gingival epithelial cells (HGEs) were purchased (Bluefbio, Shanghai, China). Both two types of cells were incubated in complete culture medium (Sigma Aldrich, Missouri, USA) containing 10% FBS and antibiotics (streptomycin 100mg/mL and penicillin 100 U mL ^-1^ (Sigma Aldrich, Missouri, USA) at 37℃ and 5% CO^2^ in an incubator. The medium was changed every three days. HGFs in passage 5 to 8 were used in the present experiments. HGEs in passage 1 to 2 were used in the present experiments.

**Effects of biofilm supernatants on production of IL-6 and TNF-𝛼 by** **Enzyme-linked Immunosorbent Assay (ELISA)**

To investigate the anti-inflammatory effects of est816 after HGFs and HGEs stimulated by *A. actinomycetemcomitans* biofilm supernatant, ELISA kits (Solarbio, Beijing, China) were conducted to determine the protein levels of interleukin-6 (IL-6) and tumor necrosis factor-𝛼 (TNF-𝛼) in cell supernatants. Briefly, cells with a density of 5× 10^6^ cell well^-1^ were seeded at 6-well plates and cultured overnight to reach 80% confluent. Then, the cells were stimulated by supernatants of *A. actinomycetemcomitans* biofilm (*A. actinomycetemcomitans* group) and of biofilm pre-treated with 12U ml^-1^ of est816 (est816+ *A. actinomycetemcomitans* group), and the supernatants were collected at 3 h and 12 h, respectively. The cells without a stimulation were served as control. Then, the 100 𝜇L supernatant was added to each well of reaction plate and incubated for 40 min at 37^∘^C. Then the ELISA plate was washed 5 times and incubated with 50 𝜇L biotinylated-antibodies for 20 min. This was followed by washing as aforementioned, 100 𝜇L enzyme conjugate was added for 10 min at 37∘C. Next, 100 𝜇L TMB solution was added and protected from light for 15 min at 37∘C prior to the addition of 100 𝜇L stopping solution after being washed extensively. The absorbance was read using a microplate reader at 450nm within 30 min. Eight-point standard curves were created based on IL-6, TNF-𝛼 standards in order to quantify the levels of L-6 and TNF-𝛼 in cell culture supernatants, and the concentrations of IL-6, TNF-𝛼 were expressed as pg ml^-1^.

**Effects of est816 against the** **progression of periodontitis in rats**

**Model for Experimental Periodontitis and Treatments**

Rats were anesthetized by intraperitoneal injection of xylazine hydrochloride (Rompun, Istanbul, Turkey) (10 mg kg ^-1^) and ketamine hydrochloride (Ketalar, Istanbul, Turkey) (40 mg kg^-1^), and then a sterile nylon thread ligature was tied to the cervical area of the bilateral maxillary first molars to establish a periodontitis model. After that, the gingival pouch of ligation area in the *A. actinomycetemcomitans* and est816 + *A. actinomycetemcomitans* groups were treated with 0.15 mL of 1.0×10^7^ CFU mL^-1^ *A. actinomycetemcomitans* suspensions and a mixture of *A. actinomycetemcomitans* and 12 U ml^-1^ est816 every 4 days, respectively. Rats placed without a ligature on maxillary first molars on both sides served as controls and were treated with the same amount of physiological saline solution. At 1 and 2 months of treatment, the rats in each group were euthanized.

**Histopathological and Immunohistochemical Analysis**

The researchers who performed the micro-CT measurements got trained and calibrated. The main technical and quality control personnel is Tao Zhou, staff from Xi 'an Jiaotong University [2]. Another technician was trained by Zhou. The intra-examiner reliability was assessed by repeated measurements at the site level, with the correlation coefficient of 0.960 (95% confidence intervals: 0.902–0.984). The inter-examiner reliability was assessed by the comparison within the two reference examiners, with the correlation coefficient of 0.870 (95% confidence intervals: 0.790–0.922). At 2 months after decalcification, the maxillae were decalcified with 10% EDTA, and the tissues from the first to the second molars were imbedded in paraffin to obtain 4-μm continuous slices in the sagittal plane prepared for hematoxylin and eosin (HE) staining and immunohistochemistry staining.

**Histopathological Assay**

HE staining (5 specimens/group) was performed to assess the integrity and inflammatory response of alveolar bone and cementum under histological observation by an optical microscope (OLYMPUS AX80, Olympus Co., Tokyo, Japan).

**Immunohistochemical Analysis**

To evaluate the levels of matrix metalloproteinase-9 (MMP-9), osteoprotegerin (OPG) and receptor activator of the NF-κB ligand (RANKL) in periodontal tissue from each group, each tissue slice (5 specimens/group) was deparaffinized and rehydrated and then treated with 0.05% trypsin to extract antigen. After washing with PBS, the sections were quenched with 3% hydrogen peroxide for endogenous peroxidase blockade and incubated with primary antibodies (Santa Cruz Biotechnology, INTERPRISE, Brazil): MMP-9, 1:400; OPG, 1:400 and RANKL, 1:400 overnight at 4 °C for 2 h. Then, slices were washed and incubated with a streptavidin-HRP-conjugated secondary antibody (Biocare Medical, CA, USA) for 30 min. A colorimetric-based detection kit (Biocare Medical, CA, USA) was used to visualize the immunoreactivity to MMP-9, OPG and RANKL, and the positive staining areas were evaluated by ImageJ software (Motic Medical 6.0; Motic, Xiamen, China).

**Results**

**Degradation of AHLs by est816****.** HPLC assays were used to analyze the degradation efficiency of C_8_-HSL, C_12_-HSL and OC_8_-HSL by est816. est816 possessed great AHL-degrading activity, and the hydrolysis rates of C_8_-HSL, C_12_-HSL and OC_8_-HSL were 66.79%, 41% and 66.11%, respectively.

**Supplementary Figures and Tables**

**Supplementary Table 1.** Polymerase chain reaction primers of *A. actinomycetemcomitans*


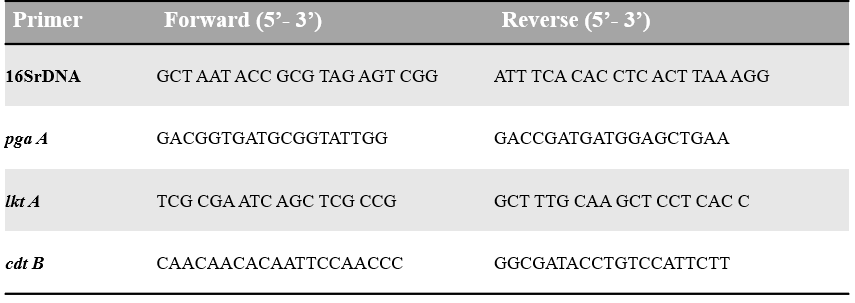


**Supplementary Figure 1.** **Schematic diagram for measuring alveolar crest absorption between maxillary first and second molars.** (A) The length of the yellow line indicates the height of alveolar crest absorption. (B) The sites for measuring bone loss at root bifurcation represented by five red points. (C) The ROI region exhibited by green area.


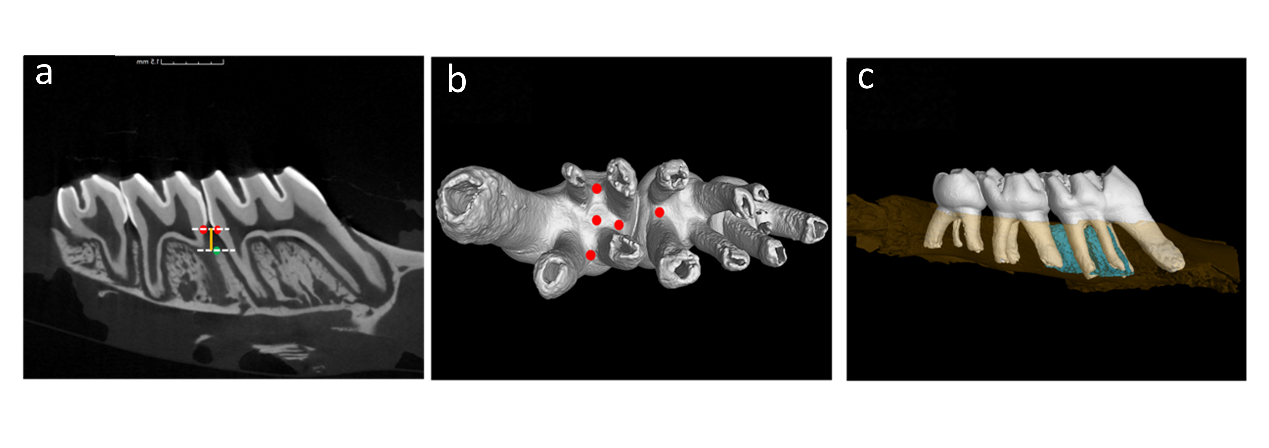


**References**

[1] Fan XJ, Liang MJ, Wang L, Chen R, Li H, Liu XL. Aii810, a novel cold-adapted n-acylhomoserine lactonase discovered in a metagenome, can strongly attenuate pseudomonas aeruginosa virulence factors and biofilm formation. *Frontiers in Microbiology*. 2017; 8:1950.

[2] Zhou T, Zhao CM, Chai ZG et al. Microscopic CT observation of root and root canal system of mandibular second permanent molar. *Chinese Journal of Conservative Dentistry*. 2014; 24:469-472+480.
